# Supplementary material for: Peroxisomal PEX7 Receptor Affects Cadmium-Induced ROS and Auxin Homeostasis in Arabidopsis Root System
Source: Antioxidants (Basel). 2021 Sep 20;10(9):1494. doi: 10.3390/antiox10091494 (PMC8471170; doi:10.3390/antiox10091494)
Supplement: Supplementary file 1 [file antioxidants-10-01494-s001.zip › antioxidants-1338333-supplementary.pdf]

Supplementary Materials

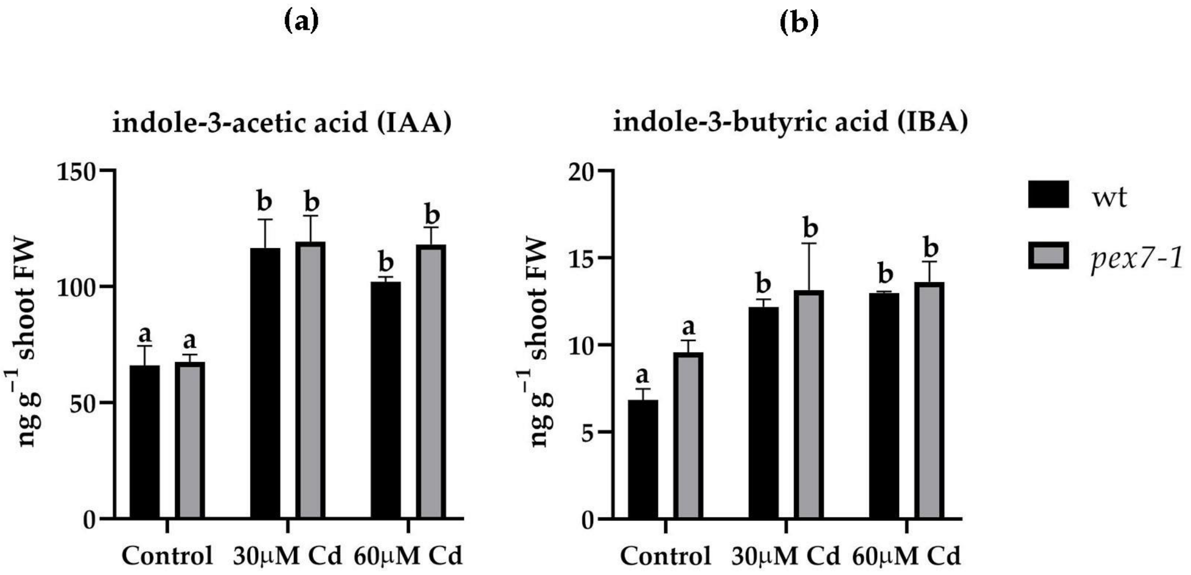

Figure S1: Mean values (±SE) from three technical replicates of IAA (**a**) and IBA (**b**) content (ng g^{-1} FW) in shoots of wt and *pex7-1* seedlings not exposed (Control) or exposed to 30 μM $CdSO_4$ or 60 μM $CdSO_4$. Columns labelled with different letters show statistical differences for at least $p < 0.05$ among treatments within the same genotype and between genotypes within the same treatment. Columns labelled with the same letter, or no letter are not statistically different ($p > 0.05$). Data from the first biological replicate are reported.

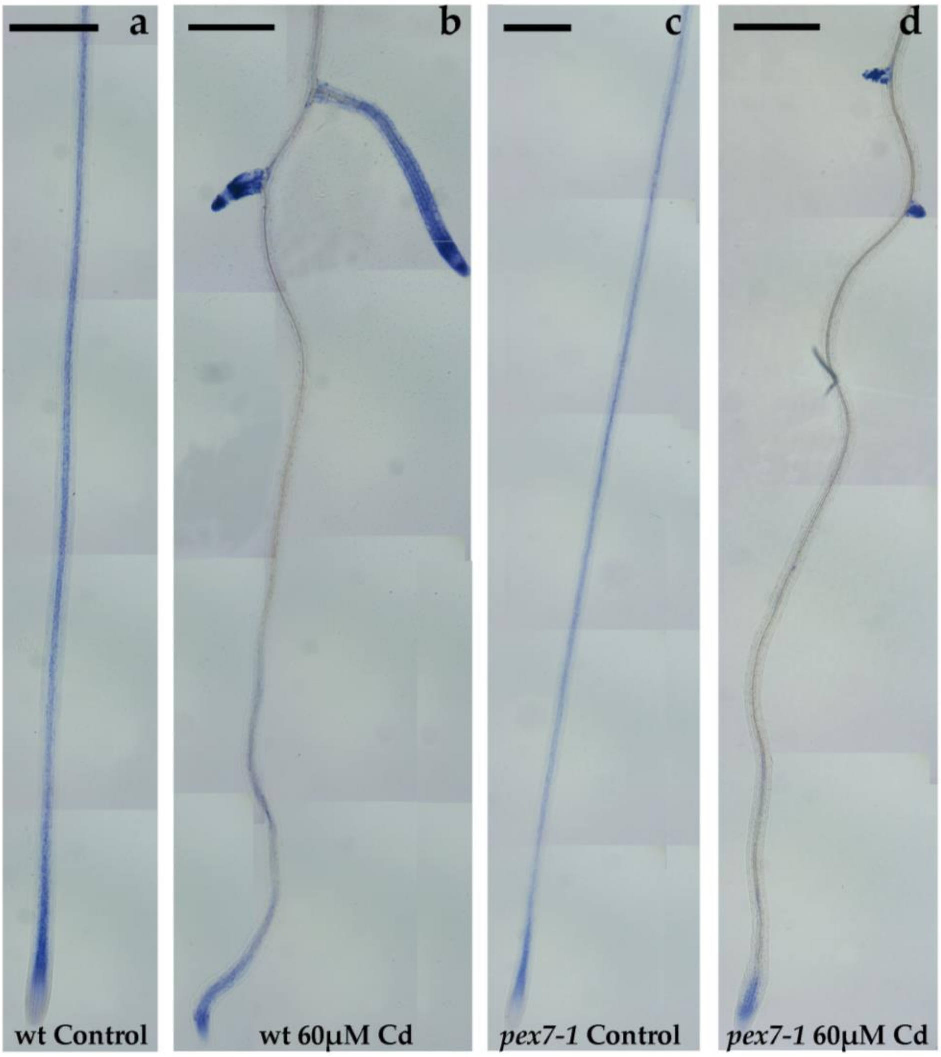

Figure S2: NBT histochemical analysis showing $O_2^{\bullet -}$ staining in the primary and lateral roots of wt (**a**,**b**) and *pex7-1* (**c**,**d**) seedlings grown in the absence ((**a**,**c**), Control) or in the presence 60 μM $CdSO_4$ (60 μM Cd) (**b**,**d**). Images were acquired with an Axio Imager M2 microscope and image tiles scanning were performed with an Axiocam 105 camera using Zen pro 2.5 software. Bars = 500 μm.
